# Electrical and Optical Properties of Indium and Lead Co-Doped Cd_0.9_Zn_0.1_Te

**DOI:** 10.3390/ma14195825

**Published:** 2021-10-05

**Authors:** Yasir Zaman, Vineet Tirth, Nasir Rahman, Amjad Ali, Rajwali Khan, Ali Algahtani, Kashif Irshad, Saiful Islam, Tao Wang

**Affiliations:** 1 State Key Laboratory of Solidification Processing, School of Materials Science and Engineering, Northwestern Polytechnical, University, Xi’an 710072, China; yasir.zaman@uos.edu.pk (Y.Z.); taowang@nwpu.edu.cn (T.W.); 2 Department of Physics, University of Sargodha, University Road Sargodha, Punjab 40100, Pakistan; 3 Mechanical Engineering Department, College of Engineering, King Khalid University, Abha 61411, Asir, Saudi Arabia; vtirth@kku.edu.sa (V.T.); alialgahtani@kku.edu.sa (A.A.); 4 Research Center for Advanced Materials Science (RCAMS), King Khalid University, P.O. Box No. 9004, Abha 61413, Asir, Saudi Arabia; 5 Department of Physics, University of Lakki Marwat, Lakki Marwat 28421, Pakistan; nasir@ulm.edu.pk; 6 Interdisciplinary Research Center for Renewable Energy and Power Systems (IRC-REPS), King Fahd University of Petroleum & Minerals, Dhahran 31261, Saudi Arabia; Kashif.irshad@kfupm.edu.sa; 7 Civil Engineering Department, College of Engineering, King Khalid University, Abha 61411, Asir, Saudi Arabia; sfakrul@kku.edu.sa

**Keywords:** I-V measurement, Hall measurement, IR Transmittance, IR Microscopy, PL

## Abstract

We have investigated the electrical and optical properties of Cd_0.9_Zn_0.1_Te:(In,Pb) wafers obtained from the tip, middle, and tail of the same ingot grown by modified vertical Bridgman method using I-V measurement, Hall measurement, IR Transmittance, IR Microscopy and Photoluminescence (PL) spectroscopy. I-V results show that the resistivity of the tip, middle, and tail wafers are 1.8 × 10^10^, 1.21 × 10^9,^ and 1.2 × 10^10^ Ω·cm, respectively, reflecting native deep level defects dominating in tip and tail wafers for high resistivity compared to the middle part. Hall measurement shows the conductivity type changes from n at the tip to p at the tail in the growth direction. IR Transmittance for tail, middle, and tip is about 58.3%, 55.5%, and 54.1%, respectively. IR microscopy shows the density of Te/inclusions at tip, middle, and tail are 1 × 10^3^, 6 × 10^2^ and 15 × 10^3^/cm^2^ respectively. Photoluminescence (PL) spectra reflect that neutral acceptor exciton (A^0^,X) and neutral donor exciton (D^0^,X) of tip and tail wafers have high intensity corresponding to their high resistivity compared to the middle wafer, which has resistivity a little lower. These types of materials have a large number of applications in radiation detection.

## 1. Introduction

Cadmium Zinc Telluride (CZT) is an important and the most promising semiconductor material having many applications in various fields because of its excellent optical and electrical properties suitable for X-rays and ɤ-rays detection, for example, medical imaging, security inspection, high energy physics, and nuclear spectroscopy [1,2,3,4,5,6,7]. Generally, the detector operating at room temperature acquires semiconductor materials to have a larger bandgap energy with high resistivity and a low leakage current. However, during the growth of CZT, Te precipitate, cadmium vacancies, and other defects will be formed. The resistivity of the crystal becomes lower, even below 106 Ω cm, which makes it unsuitable for the detector’s operation [8]. The Cadmium vacancies (V_Cd_) also change the conductivity type. Another, the CZT doped with lead (CZT:Pb), shows higher resistivity over 10^9^ Ω·cm because of the compensation of V_Cd_ by dopants [9]. High resistivity CZT are generally grown by the modified vertical Bridgman method [10]. Different dopants have been used to improve CZT crystal quality in order to minimize the defects and to improve its resistivity. Indium (In) and In:Al doped CZT have shown a shallow donor below the conduction band, while Al-doped CZT shows shallow acceptors above the valence band [11]. We have doped CZT crystals with In and Pb, which acts as a shallow level and deep level donors respectively, to compensate Cd vacancies and to improve the performance of crystals. Hall effect measurement systems measures Hall voltage, Hall coefficient, mobility, carrier concentration and type of semiconductors materials, whereas current-voltage (I-V) measurement gives us the most authentic curve, from which we can find the resistivity of the material. Infrared Transmittance also has a relation with CZT crystalline quality [10]. Infrared microscopy is the tool to measure Te inclusions and defects density in the crystal. Photoluminescence (PL) is a non-destructive, sensitive and non-contact tool used to characterize donors and acceptor impurities and other intrinsic defects in CZT crystals [12,13].

In this research paper, the co-doped CZT:In,Pb has been characterized by using I-V, Hall measurement, Infrared Transmittance, Infrared microscopy and PL spectra. The wafers have been chosen from different parts of the ingot to know the effect of co-dope on CZT crystal quality.

## 2. Materials and Methods

In the current work, the co-doped Cd_0.9_Zn_0.1_Te:(In,Pb) was grown in our laboratory by using a modified vertical Bridgman method (MVB). Growth rate was 1 mm/h and temperature gradient was 10 k/cm. The ingot was then cut into wafers with the size of (7 × 7 × 2) mm^3^. The wafers were taken from tip, middle and tail of the ingot and were mechanically polished with magnesium oxide (MgO), then etched with methanol of 2% Br-MeOH for 30 s, rinsed and kept in acetone for some time for analysis. Concentration of indium and lead was 10 ppm and 2 ppm respectively [13].

The electrical properties were analyzed by using an Agilent 4155C setup at room temperature (RT) by making the gold coating on the surfaces of samples. The resistivity was measured by a Van der pauw method based on Hall effect, through which the type of material, charge carrier mobility and charge carrier concentration were obtained at RT. In the experiments, small indium contacts at the four corners of all the samples were used. Te inclusions were characterized by IR transmission microscopy using Micronviewer 7290A, while IR transmittance was tested by a Nicolet Nexus 670 spectrometer in the range of 500 to 4000 cm^−1^. The optical properties were characterized by PL, in which the samples were attached to a copper finger in a close cryostat with grease and the temperature was cold down to 10 K. An Argon ion laser with a wavelength of 488 nm was used to excite the spectra. A Triax 550 tri-grating monochromator with a photomultiplier tube (PMT) possessing a spectral resolution of better than 0.3 nm was connected to the system to collect the spectra emitted from the samples.

## 3. Results

### 3.1. Electrical Properties of Co-Doped CdZnTe:(In,Pb)

**Figure 1:**
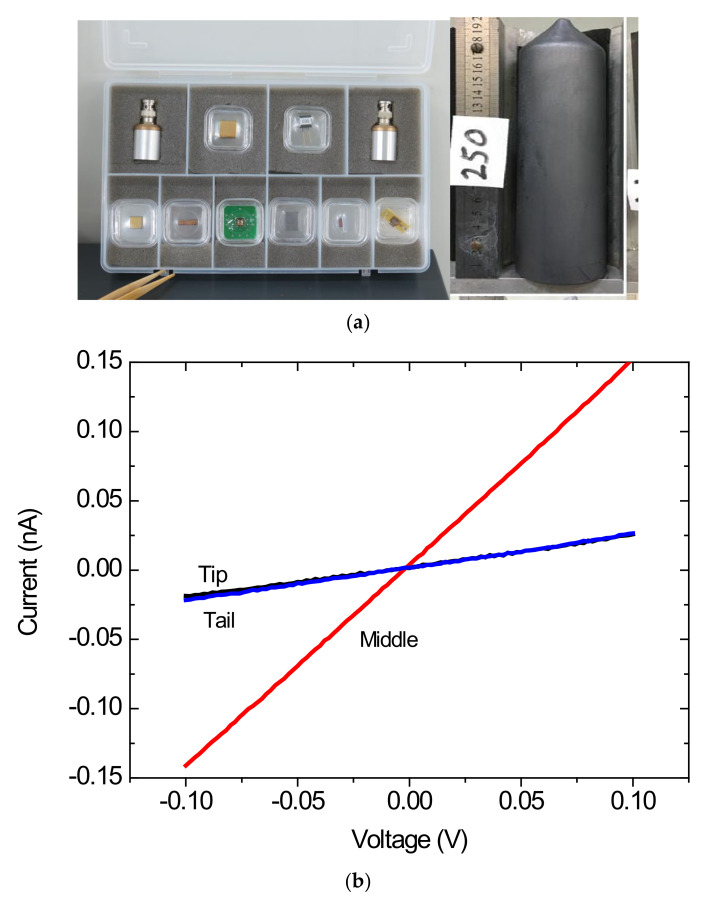
(**a**) Configuration of setup i-e holder, wafer size and Ingot CZT:(In,Pb)/Au wafers. (**b**) The I-V Curves of CZT:(In,Pb)/Au wafers from different parts of the ingot.

Figure 1a shows the holder, wafer size and Ingot grown of CZT:(In,Pb) while Figure 1b shows the I-V curves of the three wafers from different parts of the ingot. The results show a linear relationship between I and V, following Ohm’s Law. The resistivity of the tip, middle and tail wafers are 1.8 × 10^10^, 1.21 × 10^9^, and 1.2 × 10^10^ Ω·cm respectively. Native Deep traps were dominated at the tip and tail wafers, showing a higher resistivity than the middle part.

Like CdTe [14], CdZnTe is also a semi-insulating material with nonuniform conductivity [15].

Therefore, the high resistivity implies that the densities of twins, Te inclusions and dislocations are relatively high in the initial part of the ingot due to the supercooling effect on the processing of the ignot. The conductivity type of CdZnTe:(In,Pb) is changed from n to p as we go from tip to tail as shown in Table 1. The carrier concentration is also pining the Fermi level near the center of the bandgap. Hall mobility, carrier concentration, and resistivity of the wafers are also shown in Table 1.

### 3.2. IR Transmittance

**Figure 2:**
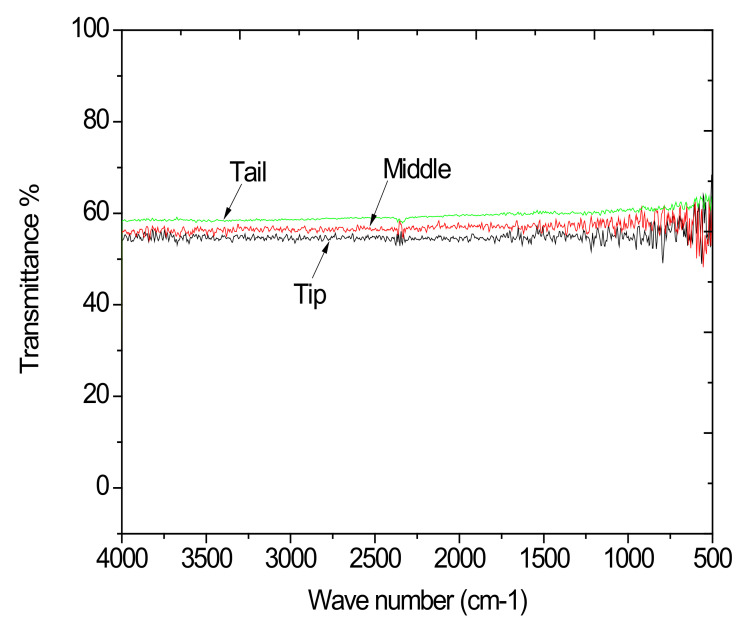
IR Transmittance spectra of co-doped CdZnTe:(In,Pb) of tip, middle and tail wafers at room temperature.

CZT ingot is transparent at IR wavelengths, but some defects in the crystal may decrease the IR transmission. Therefore, the room temperature IR transmission spectra are usually used to evaluate the quality of the crystal. In this paper, the typical IR transmission spectra with the wavelength in the range of 500–4000 cm^−1^ for the as-grown crystal are shown in Figure 2.

The maximum theoretical transmittance for CZT is about 63% [16] by using Equation (1).

T% = [(1 − *R*) ^2^/1 − *R*^2^ exp^−α*t*^] (1)

where α is absorbance coefficient, *t* is thickness of the wafer, *R* = (1 − *n*) ^2^/(1 + *n*)^2^, *n* is refractive index.

Figure 2 shows that the IR Transmittances of tail, middle and tip wafers are about 58.3%, 55.5% and 54.1% respectively in the range between 4000–500 cm^−1^ in descending order. In IR transmittance and variation in Te inclusion concentration is inconsistent; the basic reason for thus is because from tip to tail there is Te segregation, resulting in a different content of Te-rich in front of the growth interface.

It is obvious that the sample with the lowest Te inclusion has the highest IR transmission. Therefore, the Te inclusion is regarded as the main defect affecting the IR transmission besides the lattice absorption. Zaman et al. [13] have calculated the theoretical transmission of this crystal as 65%, and in this paper, sample 2 shows a high IR transmission of 58%, which is near the theoretical limit. Only, the number of Te/Inclusion does not influence transmittance and there is no direct relationship between them. The density of Te/inclusion can decide only the higher transmittance of tail. The size of Te inclusion in the tail may be smaller. The size of Te inclusion is much influenced by the transmittance and not density; therefore, it seems to be consistent.

### 3.3. IR Microscopy

**Figure 3:**
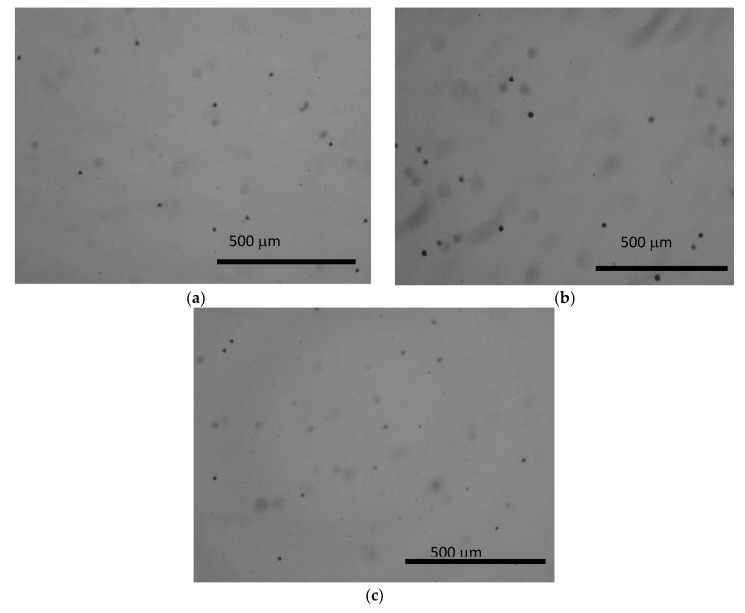
Typical IR Microscopy of CZT:In,Pb. (**a**) tip wafer, (**b**) middle wafer, (**c**) tail wafer.

Figure 3 shows that Te inclusions are uniformly distributed throughout the samples, with the size of less than 20 µm in diameter [17]. The total densities of the respective wafers from tip, middle and tail are 1 × 10^3^, 6 × 10^2^ and 15 × 10^3^/cm^2^ respectively. It shows that the density of Te inclusions in the tail of the ingot is higher than those from other parts, which could be attributed to the instability of the solid–liquid interface during crystal growth [18]. This indicates that Te inclusions/precipitates are prone to enrichment in the initial region (tip) and the end of the ingot.

Because the initial part of the ingot is a supercooled area, the Te excess of 10 at.% was assumed to increase the degree of supercooling. Consequently, the initial region was characterized by an anomalous segregation in relation to the more Te inclusions/precipitates [17,18]. With the progressing of the growth, the relative extent of the excess Te was increasing by more and more, resulting in the enriching of Te inclusions at the end of the ingot.

### 3.4. PL Measurements of Co-Doped (CZT:In,Pb)

**Figure 4:**
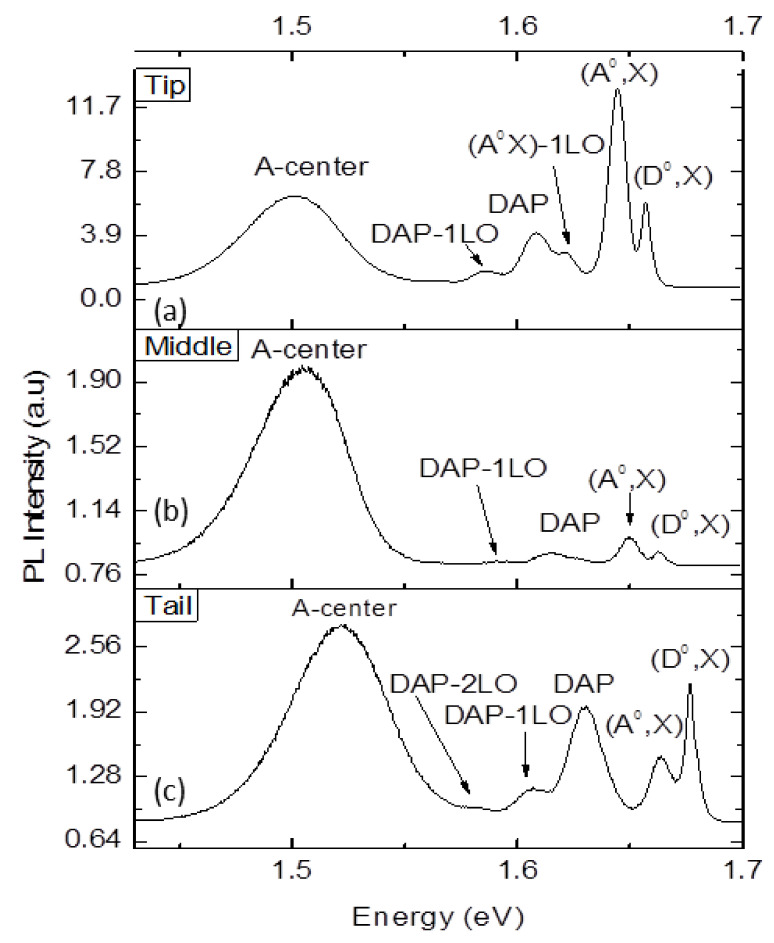
PL spectrum of co-doped CdZnTe: (In,Pb) crystal at 10 K. (**a**) tip wafer (**b**) middle wafer (**c**) tail wafer.

Figure 4 shows typical PL spectra of tip, middle and tail wafers at 10 K. Several peaks are observed in each wafer. The tip wafer shown in Figure 4a has many transitions over different energy ranges. A-center peak is observed at 1.501 eV, while the donor acceptor pair (DAP) is at the energy of 1.609 eV. One Longitudinal optical (1LO) phonon replica at the energy of 1.585 eV is observed, which is very weak. Similarly, neutral acceptor-bound excitons (A^0^,X) at the energy of 1.644 eV with high intensity and 1LO replica at 1.622 eV are observed. Neutral donor bound excitons (D^0^,X) at the energy of 1.656 eV has very low energy compared to (A^0^,X) [19,20]. For the middle wafer, as shown in Figure 4b, A-center with the energy of 1.504 eV and DAP, DAP-1LO, (A^0^,X) and (D^0^,X) with the energies of 1.616, 1.590, 1.649 and 1.633 eV were observed. The intensity of all the peaks is very low except for (A^0^,X). As shown in Figure 4c, the tail wafer has an A-center at 1.522 eV, DAP at 1.629 eV, DAP-1LO at 1.607 eV, DAP-2LO at 1.582 eV, (A^0^,X) at 1.663 eV and (D^0^,X) at 1.677 eV. An intensity ratio of (A^0^,X)/(D^0^,X) for tip, middle and tail wafers is 2.163, 1.089 and 0.674, respectively.

## 4. Conclusions

Our objective was to obtain a material that has a large number of applications in radiation detection. The (CZT:In,Pb) ingot was grown in our laboratory using the modified vertical Bridgman method (MVB). The resistivity values of 1.8 × 10^10^, 1.21 × 10^9^ and 1.2 × 10^10^ Ω·cm for the tip, middle and tail wafers, respectively, showed complete agreement with Ohm’s law measured from I-V curves. The native deep traps introduced in the tip and tail wafers increased their corresponding resistivity values to a higher degree than that of the middle wafer. The maximum value of the Hall mobility of 9.24 × 10^2^ cm^2^/V·S (Tip), the carrier concentration of 6.8 × 10^9^ cm^−3^ (Middle) and P-type conductivity for (Tail) type sample were measured through Hall measurements, which are considered acceptable for the detector’s fabrication. Furthermore, the IR Transmittance values vary from 58.3%, 55.5%, and 54.1% for the wafers from tail to tip. An IR Microscopy shows the uniform distribution of Te precipitates throughout the samples. PL spectra at 10 K show that the intensity of (A^0^,X) and (D^0^,X) for the wafers from the tip and tail are higher than the middle. The quality of co-doped (CZT:In,Pb) crystals was comparable to that of In-doped crystals from the characterization of optical and electrical properties, which are the key technology in various fields, such as medical imaging and security inspection. Moreover, the CZT-based materials are best for radiation detectors and gamma spectrometers, which are highly sensitive room temperature radioactivity monitors

## Figures and Tables

**Table 1:** Hall and I-V measurement results of Tip, Middle and Tail wafers of co-doped CdZnTe:(In,Pb) crystals.

Sample Names	Conductivity Type (N or P)	Hall Mobility (cm^2^/V·S)	Carrier Conc. (cm^−3^)	Resistivity (Ω·cm)
Tip	N	9.24 × 10^2^ cm^2^/V·S	6.45 × 10^7^ cm^−3^	1.8 × 10^10^ Ω·cm
Middle	N	11.88 × 10^2^ cm^2^/V·S	6.8 × 10^9^ cm^−3^	1.21 × 10^9^ Ω·cm
Tail	P	13.7 cm^2^/V·S	6.78 × 10^8^ cm^−3^	1.2 × 10^10^ Ω·cm

## Data Availability

The data presented in this study are available on request from the corresponding authors.
